# Author Correction: SARS-CoV-2 infection induces inflammatory bone loss in golden Syrian hamsters

**DOI:** 10.1038/s41467-022-30952-x

**Published:** 2022-05-31

**Authors:** Wei Qiao, Hui En Lau, Huizhi Xie, Vincent Kwok-Man Poon, Chris Chung-Sing Chan, Hin Chu, Shuofeng Yuan, Terrence Tsz-Tai Yuen, Kenn Ka-Heng Chik, Jessica Oi-Ling Tsang, Chris Chun-Yiu Chan, Jian-Piao Cai, Cuiting Luo, Kwok-Yung Yuen, Kenneth Man-Chee Cheung, Jasper Fuk-Woo Chan, Kelvin Wai-Kwok Yeung

**Affiliations:** 1 Department of Orthopaedics and Traumatology, School of Clinical Medicine, Li Ka Shing Faculty of Medicine, the University of Hong Kong, Hong Kong S.A.R., China; 2 Shenzhen Key Laboratory for Innovative Technology in Orthopaedic Trauma, the University of Hong Kong-Shenzhen Hospital, Shenzhen, 518053 China; 3 Applied Oral Sciences & Community Dental Care, Faculty of Dentistry, the University of Hong Kong, Hong Kong S.A.R., China; 4 State Key Laboratory of Emerging Infectious Diseases, Carol Yu Centre for Infection, Department of Microbiology, School of Clinical Medicine, Li Ka Shing Faculty of Medicine, The University of Hong Kong, Pokfulam, Hong Kong Special Administrative Region China; 5 Centre for Virology, Vaccinology and Therapeutics, Hong Kong Science and Technology Park, Hong Kong Special Administrative Region, China; 6 Department of Clinical Microbiology and Infection Control, The University of Hong Kong-Shenzhen Hospital, Shenzhen, Guangdong Province China; 7 Academician Workstation of Hainan Province of Hainan Medical University, and Hainan Medical University-The University of Hong Kong Joint Laboratory of Tropical Infectious Diseases, Hong Kong Special Administrative Region, China; 8 Guangzhou Laboratory, Guangzhou, Guangdong Province China

**Keywords:** Viral infection, SARS-CoV-2

Correction to: *Nature Communications* 10.1038/s41467-022-30195-w, published online 09 May 2022.

In this article the author name Kwok-Yung Yuen was incorrectly written as Kwok-Yong Yuen. The original article has been corrected.

